# Lower FSH With Normal Fertility in Male Mice Lacking Gonadotroph Kisspeptin Receptor

**DOI:** 10.3389/fphys.2022.868593

**Published:** 2022-04-26

**Authors:** Yaping Ma, Olubusayo Awe, Sally Radovick, Xiaofeng Yang, Sara Divall, Andrew Wolfe, Sheng Wu

**Affiliations:** ^1^ Department of Pediatrics, Johns Hopkins University School of Medicine, Baltimore, MD, United States; ^2^ Department of Pediatrics, Affiliated Hospital of Jiangnan University, Wuxi, China; ^3^ Department of Cellular and Molecular Physiology, Johns Hopkins University School of Medicine, Baltimore, MD, United States; ^4^ Department of Pediatrics, Rutgers University Medical School, New Brunswick, NJ, United States; ^5^ Department of Cardiovascular Sciences, Center for Metabolic Disease Research, Temple University School of Medicine, Philadelphia, PA, United States; ^6^ Department of Pediatrics, University of Washington, Seattle’s Children’s Hospital, Seattle, United States

**Keywords:** kisspeptin receptor, GnRH, pituitary, reproduction, LH, FSH, puberty

## Abstract

The kisspeptin receptor, crucial for hypothalamic control of puberty and reproduction, is also present in the pituitary gland. Its role in the pituitary gland is not defined. Kisspeptin signaling *via* the Kiss1r could potentially regulate reproductive function at the level of pituitary gonadotrope. Using Cre/Lox technology, we deleted the *Kiss1r* gene in pituitary gonadotropes (PKiRKO). PKiRKO males have normal genital development (anogenital distance WT: 19.1 ± 0.4 vs. PKiRKO: 18.5 ± 0.4 mm), puberty onset, testes cell structure on gross histology, normal testes size, and fertility. PKiRKO males showed significantly decreased serum FSH levels compared to WT males (5.6 ± 1.9 vs. 10.2 ± 1.8 ng/ml) with comparable LH (1.1 ± 0.2 vs. 1.8 ± 0.4 ng/ml) and testosterone levels (351.8 ± 213.0 vs. 342.2 ± 183.0 ng/dl). PKiRKO females have normal puberty onset, cyclicity, LH and FSH levels and fertility. Overall, these findings indicate that absence of pituitary Kiss1r reduces FSH levels in male mice without affecting testis function. PKiRKO mice have normal reproductive function in both males and females.

## Introduction

Kisspeptin and its G-protein coupled receptor (KISS1R/GPR54) have a critical role in regulating the hypothalamic control of puberty and reproduction in all mammalian species tested to date (de Roux et al., 2003; Funes et al., 2003; Gottsch et al., 2004; Messager et al., 2005; Teles et al., 2008; Dror et al., 2013; Witham et al., 2013; Martins Trevisan et al., 2020; Padda et al., 2021). In rodents, kisspeptin is highly expressed in hypothalamic anteroventral periventricular nucleus (AVPV) and arcuate nucleaus regions (ARC) (Seminara, 2005; Clarkson and Herbison, 2006; Martins Trevisan et al., 2020). *Kiss1r* is expressed in hippocampus, septum, rostral preoptic area (rPOA), anteroventral nucleus of the thalamus, and throughout the arcuate nucleus (Kauffman et al., 2007; Herbison et al., 2010; Higo et al., 2016; Ozaki et al., 2019). Conditional knock-out studies defined the crucial role of *Kiss1r* receptor signaling in the GnRH neuron for normal reproductive development and fertility (Clarkson et al., 2009; Novaira et al., 2014). Kiss1r is also expressed in much smaller amounts in the pituitary (Gutiérrez-Pascual et al., 2007; Witham et al., 2013) as well as in the placenta, liver, pancreas and intestine (Brown et al., 2008; Song et al., 2014; de Pedro et al., 2015).

The pituitary incorporates diverse cues and signals to affect gonadotropin (luteinizing hormone and follicle stimulating hormone) release including estradiol (Singh et al., 2009), testosterone (Ramaswamy et al., 2007), progesterone (Girmus and Wise, 1992), metabolic hormones such as insulin (Williams et al., 1987; Brothers et al., 2010). Hypothalamus derived factors including GnRH travel from the hypothalamic median eminence to the pituitary using the portal venous system. Kisspeptin is released to the circulation and has potential to activate KISS1R signaling cascades of pituitary cells.

Kisspeptin increases gonadotropin gene expression in mouse primary pituitary cells in culture (Witham et al., 2013). *Kiss1r* expression is enhanced in the pituitary of female mice during the estradiol-induced LH surge (Clarkson et al., 2008), suggesting a possible role for kisspeptin as part of the constellation of regulatory inputs to the pituitary gonadotroph required for the preovulatory release of gonadotropins. We sought to examine the physiological role of pituitary gonadotroph kisspeptin receptor *in vivo* using a novel kisspeptin receptor knockout mouse (PKiRKO mouse). In male mice with absence of the gonadotroph kisspeptin receptor, FSH levels are lower than wild type mice, gonads have normal size and hormone production, and fertility is normal.

## Materials and Methods

### Generation of Gonadotrope-Specific Kiss1R Knockout Mice (PKiRKO)

To generate pituitary Kiss1 receptor (Kiss1R) knockout (PKiRKO) mice, we crossed *Kiss1r* heterozygous (fl/wt) female mice (Novaira et al., 2014) with αGSU transgenic (αCre+/-) male (Naik et al., 2006; Brothers et al., 2010) mice. The αGSU transgenic (αCre+/-) effectively deletes floxed genes specifically in pituitary gonadotrophs and thyrotropes (Brothers et al., 2010). F1: female mice (Kiss1R fl/wt; αCre+) and male mice (Kiss1R fl/fl; αCre-) were crossed to produce PKiRKO mice (Kiss1R fl/fl; αCre+). Litter mates (*Kiss1r* fl/wt; αCre- and *Kiss1r* fl/fl; αCre-) were used as controls (referred as WT). DNA was extracted as described previously (Wolfe et al., 2008). Genotyping primers were designed to detect the presence of *αCre* and the floxed allele, WT allele, or knockout allele of *Kiss1r*: P1 sense (located in exon 1) and P3 antisense (located in exon 3). PCR of tail genomic DNA using primers P1 and P3 will amplify a 2096-bp amplicon to indicate the floxed Kiss1r allele and 1882-bp amplicon to indicate the WT allele. Genomic DNA obtained from the pituitary using primers P1 and P3 will amplify a 1120-bp amplicon if the sequence between the LoxP sites is excised indicating a KO allele. Primer set for *αCre* is *αCre* F: GCC​ACC​ACC​GCC​CTG​CTT​AAG​TAA; R: GCC​ACC​ACC​GCC​CTG​CTT​AAG​TAA); for *Kiss1r* is P1: CTG​GTC​GGA​AAC​TCA​TTG​GT; P3: AGA​GTG​GCA​CAT​GTG​GCT​TG.

### Animals

Adult male and female mice (>2 months old) were used in this study. All animal studies were carried out in accordance with National Institutes of Health guidelines on animal care regulations and were approved by the Animal Care and Use Committee of the Johns Hopkins University. Mice were maintained under constant conditions of light and temperature (14: 10 h light⁄dark cycle; 22 C) and were fed a normal chow and water ad libitum.

### Quantitative Real-Time PCR (qPCR)

RNA was extracted from the pituitary, liver, ovaries, uterus, testes, epididymis, adipose tissue and from two hypothalamic fragments encompassing the arcuate and the anteroventral periventricular nucleus (AVPV) (Quennell et al., 2011), using TRIzol reagent (Ambion life technologies, Carlsbad CA, United States ), according to the protocol provided by the supplier. 1µg of RNA was reverse transcribed to cDNA using an iScript cDNA kit (Bio-Rad Laboratories). Real-time qPCR was performed to determine the presence and relative expression levels of *Kiss1*r mRNA in the various tissues. Real-time qPCR was performed in duplicate using SYBR Green Master Mix (Bio-Rad Laboratories) and the CFX Connect qPCR machine (Bio-Rad Laboratories). *Kiss1r* primer sequences are Sense: CTG​CCA​CAG​ACG​TCA​CTT​TC; antisense, 5-ACA​TAC​CAG​CGG​TCC​ACA​CT. For each primer, PCR efficiency was determined by measuring a 10-fold serial dilutions of cDNA and reactions having 95 and 105% PCR efficiency were included in subsequent analyses. Relative differences in cDNA concentration between WT and PKiRKO mice were then calculated using the comparative threshold cycle (C_t_) method. To compare the difference of *Kiss1*r expression in the same tissue between WT and PKiRKO, a △C_t_ was calculated to normalize for internal control using the equation: C_t_ (Kiss1R)—C_t_ (18S). △△C_t_ was calculated: △C_t_ (PKiRKO) -△C_t_ (WT). Relative Kiss1R mRNA levels were then calculated using the equation fold difference = 2^△△^C_t_.

### Pubertal Onset and Assessment

Preputial separation (PPS) in males was assessed daily beginning after postnatal day 21 by determining whether the prepuce could be manually retracted with gentle pressure. PPS is testosterone dependent and thus is an indicator of activation of the reproductive axis in males. Puberty in rodents is dependent on body weight (Baker, 1985) hence, Body weight (BW) of PKiRKO and wild type were assessed once a week in prepuberty (day 21) through adulthood (day 49). Anogenital distance is testosterone dependent and was determined at 8 weeks of age. Wet testicular weights were determined in freshly dissected mice at 2 months of age. In females, age at vaginal opening and first estrus are two markers of puberty onset and were assessed for daily in mice after 21 days of life until achieved. Vaginal smears were obtained daily over a period of 14 consecutive days in 6- to 11-week-old mice and cellular morphology examined under microscope. BW was also recorded at the age of PPS and first estrus.

### Hormone Assays

Blood samples were collected from submandibular vein (Wu et al., 2014; Wang et al., 2019) between 9:00 and 10:00 a.m. and basal levels of serum LH and FSH were measured. LH and FSH were measured using a Milliplex MAP immunoassay (Mouse Pituitary panel; Millipore, Massachusetts) on a Luminex 200IS platform (Luminex Corporation). The assay detection limit for LH was 0.012 ng/ml and for FSH was 0.061 ng/ml. The intra-assay and interassay coefficients of variation (CV) for LH and FSH were between 5 and 9%. Testosterone levels were measured by radioimmunoassay by the University of Virginia Ligand Assay Core (Charlottesville, Virginia) and the intra-assay CV was 5% and inter-assay CV was 9%.

### Fertility Assessment

To determine whether male PKiRKO mice were fertile relative to controls, fertility was examined in WT and PKiRKO mice using a rotating mating protocol. 3 WT and 4 PKiRKO male mice were housed individually with proven fertile WT female mice for 14 consecutive days and then were separated. If a pregnancy ensued, 1 week after female gave birth, a new male was inserted. Males were rotated among the proven females. To assess fertility in females, a similar strategy was used. Females were housed individually with proven fertile WT male mice for 14 consecutive days and then separated. If a pregnancy ensued, 1 week after female gave birth, a new male was inserted, rotating among the seven proven males. The duration of the fertility study was 4 months with four rotations for each mouse.

### Data Analysis

Data were analyzed by two tailed unpaired student t tests (parametric) with normality of residuals test Shapiro-Wilk (W)) using Prism software (GraphPad Software, Inc., La Jolla, CA). All results are expressed as mean ± SEM (standard error of the mean). *p* < 0.05 was defined as statistically significant.

## Results

### Generation of Pituitary Specific *Kiss1r* Knockout Mice

**FIGURE 1:**
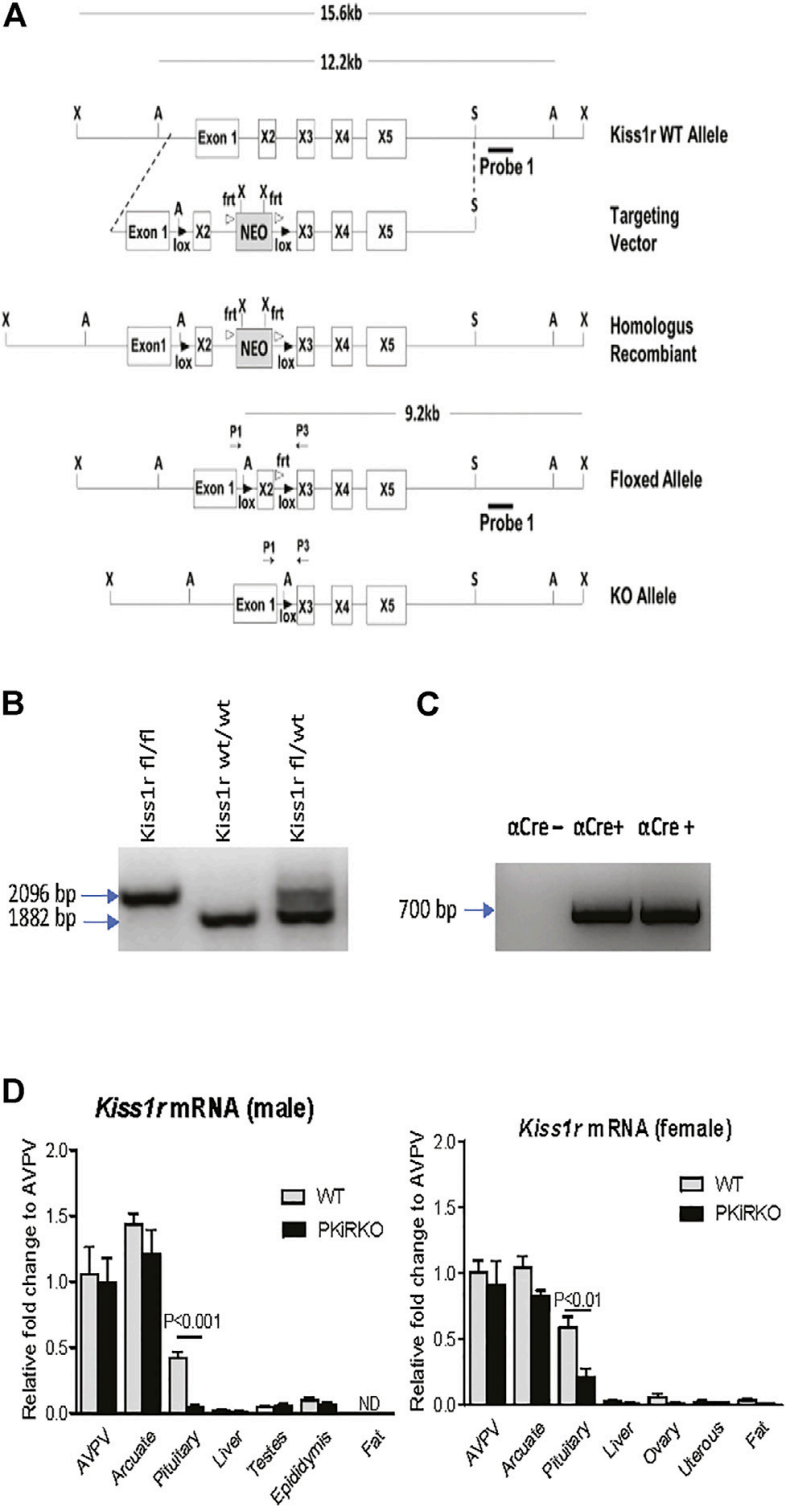
Development of the PKiRKO mouse. **(A)** schematic diagrams of constructs used to generate PKiRKO mice. Mice bearing LoxP sites flanking exon 2 of the Kiss1r were crossed with transgenic mice expressing Cre recombinase specifically in Gonadotrophs (αGSU). **(B)** Genotyping by PCR analysis of the genomic DNA produced a 2096 bp amplicon in mice with a floxed allele and an 1882 bp amplicon in kisspeptin fl/fl mice, also shown are both bands present in the heterozygous floxed-*Kiss1r*. **(C)** Lane 1-αCre^+^ transgene negative. Lane 2 & 3- αCre^+^ transgene positive mice. Amplicon size is 700 bp. **(D)** Quantitative RT-PCR analysis of Kiss1r mRNA extracted from male and female mouse tissues. *Kiss1r* was significantly reduced (87.6% for male, 63.7% for female) in the pituitary of PKiRKO (KO) mice compared with that in wild type (WT) mice, but no difference in *Kiss1r* expression was observed in other tissues. ** = *p* < 0.01. *** = *p* < 0.001. Data are means ± SEM (*n* = 8 for pituitary, *n* = 4 for other tissues).

PKiRKO (Kiss1R^fl/fl^; αCre^+^) mice were generated by Cre recombinase mediated excision of exon 2 of the *Kiss1r*, resulting in an attenuated *Kiss1r* gene in pituitary, as shown in the schematic diagram of Figure 1A. The PCR product of genotyping indicates the homozygous floxed-*Kiss1r* alleles (2096 bp) and WT alleles (1882 bp) (Figure 1B); both bands are present in the heterozygous floxed-*Kiss1r* mouse schematized by Novaira HJ *et al.* (Novaira et al., 2014). Shown in Figure 1C Lane 1 is a WT αCre-male mice, followed by two αCre + male mice in lanes 2 and 3 showed by PCR of genotyping.

### Pituitary and Tissue *Kiss1r* mRNA Expression in PKiRKO Mice

qPCR demonstrated a reduction in *Kiss1r* mRNA by 88% (Figure 1D) and 64% (Figure 1D) in the pituitary of male and female PKiRKO mice (*n* = 8), respectively, compared with WT mice (*n* = 8). In contrast, no difference in *Kiss1r* mRNA was observed between PKiRKO and control animals in other tissues including the hypothalamus, adipose tissue, liver, or gonads (*p* > 0.05, *n* = 4) (Figure 1D). There was more *Kiss1r* mRNA detected in the pituitaries of female PKiRKO mice.

### Pubertal Onset and Reproductive Function Are Normal in PKiRKO Mice

**FIGURE 2:**
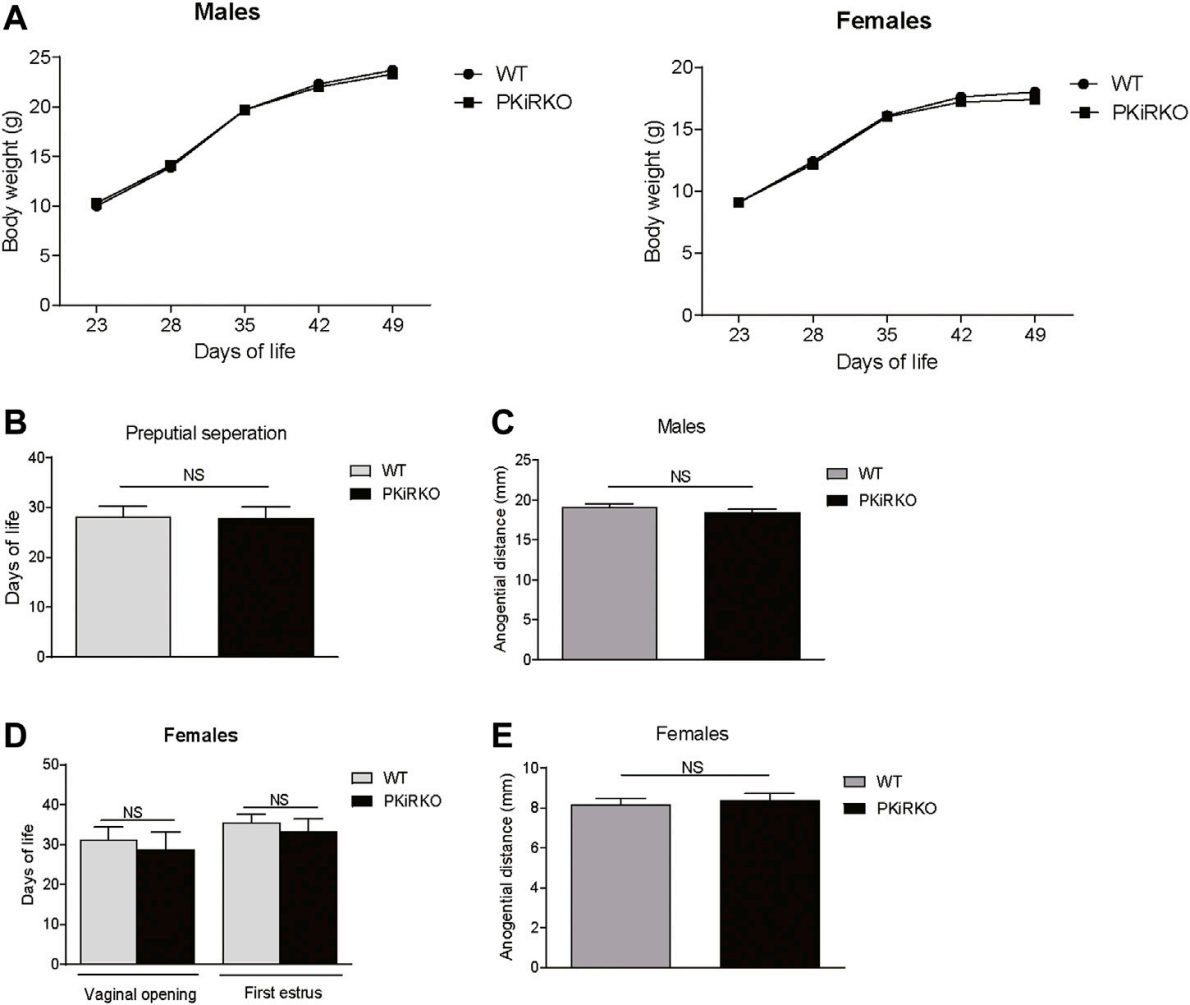
PKiIRKO mice have normal puberty onset. **(A)** Body weight change over time was not different between PKiRKO (*n* = 15) and WT (*n* = 15) male mice or female PKiRKO (*n* = 15) and WT (*n* = 15) mice. **(B)** No significant difference was observed in age of preputial separation (PPS) in males. WT (*n* = 15) PKiRKO (*n* = 15). **(C)** Anogenital distance was not different between WT and PKiRKO male mice (*n* = 6 each group). **(D)** No difference was observed in age at vaginal opening or first estrus. **(E)** Anogenital distance was not different between WT and PKiRKO female mice.

**FIGURE 3:**
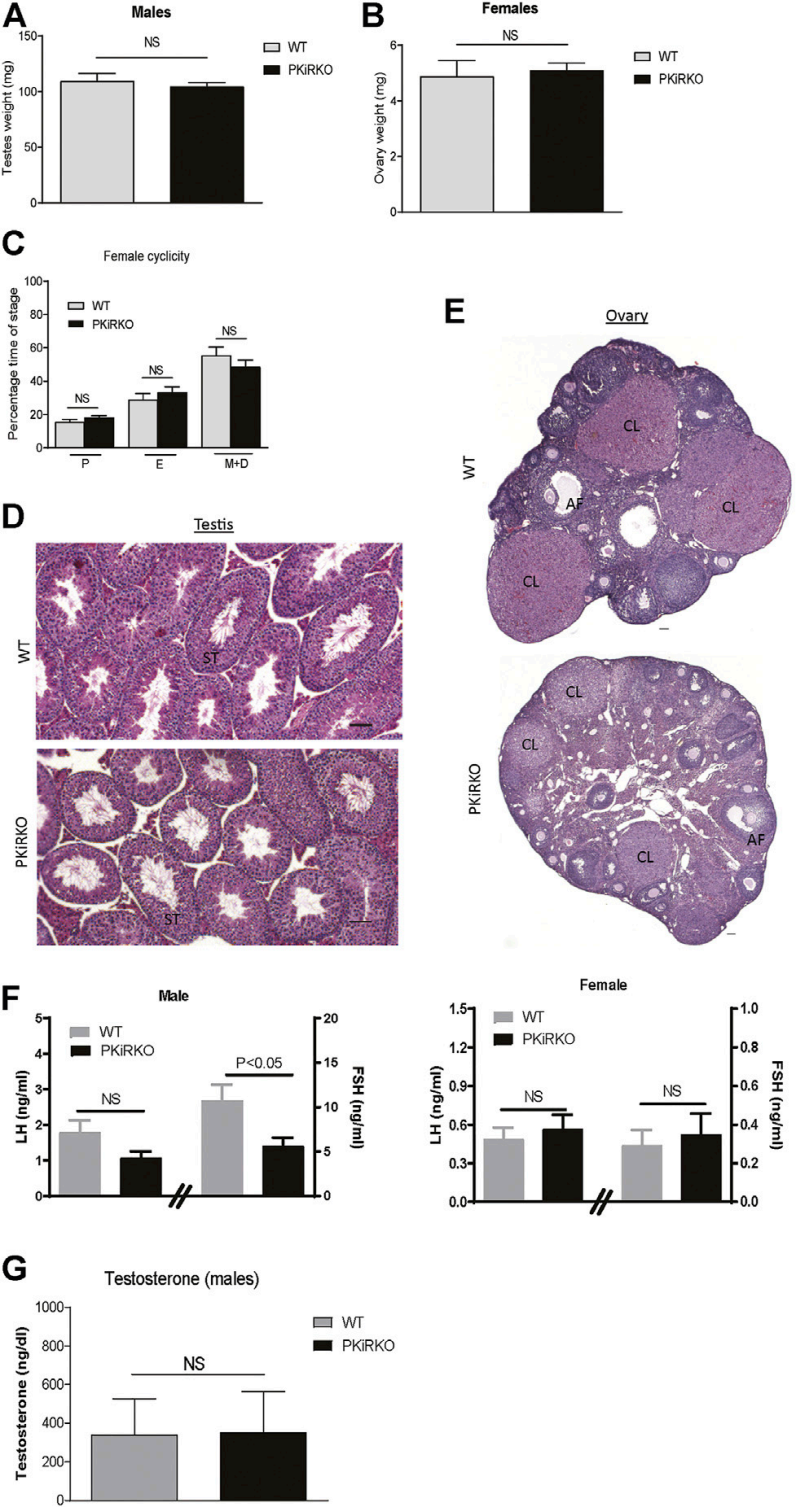
PKiRKO mice have normal reproductive function. **(A)**. Testes weight was not significantly different between PKiRKO and WT male mice (*n* = 6). **(B)** Ovary weight was not significantly different between PKiRKO and WT female mice (*n* = 6). **(C)** Vaginal cytology was examined daily over a period of 14 consecutive days in 6- to 11-week-old mice. WT and PKiRKO groups both exhibited regular estrous cycles the percentage time spent at each cycle stage did not differ between groups. *N* = 6 each group. **(D)** Testis structure between WT and PKiRKO by H&E staining. ST: seminiferous tubule. **(E)** ovary structure between WT and PKiRKO. CL: copora lutea; AF: antral follicles. There was comparable testis and ovary structure. **(F)** Morning LH (left *Y* axis and FSH (right *Y* axis) in male (left panel) and female (right panel) mice. *n* = 6, *p* > 0.05) **(G)** Testosterone levels are not different between PKiRKO and WT mice (*n* = 6 each group). NS = No significant difference.

Body weight (BW) was not different between male or female PKiRKO and WT mice (Figure 2A). In males, PPS (preputial separation) is an androgen dependent process that serves as an external sign of male puberty onset (Marty et al., 2001). No significant difference was observed age of PPS between the WT and PKiRKO groups ((28.1 ± 0.6 vs. 27.8 ± 0.6 days of life), Figure 2B), or achieved anogenital distance (Figure 2C). The age of vaginal opening and first estrus (Figure 2D), and anogenital distance (Figure 2E) was not different between female WT and PKiRKO groups. The BW at age of pubertal achievement (PPS at day 28 in males (BW: 14 ± 1 vs. 15 ± 1 g) and first estrous at day 35 in females (BW: 18 ± 1 vs. 16 ± 1 g) was not significantly different between WT and PKiRKO animals. In sexually mature mice, gonad weight is a marker of reproductive tissue function. Gonad weights were comparable between PKiRKO and WT male and female mice (Figure 3 A and B). Estrous cycling pattern was similar between PKiRKO and WT mice (Figure 3C); percent time spent at each cycle stage was not significantly different. Testes (Figure 3D) or ovary (Figure 3E) histology was not different between PKiRKO and WT mice. The morphology of seminiferous tubules were comparable between WT and PKiRKO male mice (Figure 3D). The number of sperm in the seminiferous tubules was not statistically different between WT and PKiRKO mice (data not shown).

Basal serum LH levels in PKiRKO males (1.8 ± 0.4 vs. 1.1 ± 0.2 ng/ml) or females (0.5 ± 0.1 vs. 0.6 ± 0.1 ng/ml) were not significantly different relative to WT mice, while FSH levels (10.2 ± 1.8 vs. 6.0 ± 1.9 ng/ml) were significantly lower in PKiRKO males (Figure 3F). Testosterone levels were not significantly different between WT and PKiRKO mice (Figure 3G).

### Normal Fertility in PKiRKO Mice

**FIGURE 4:**
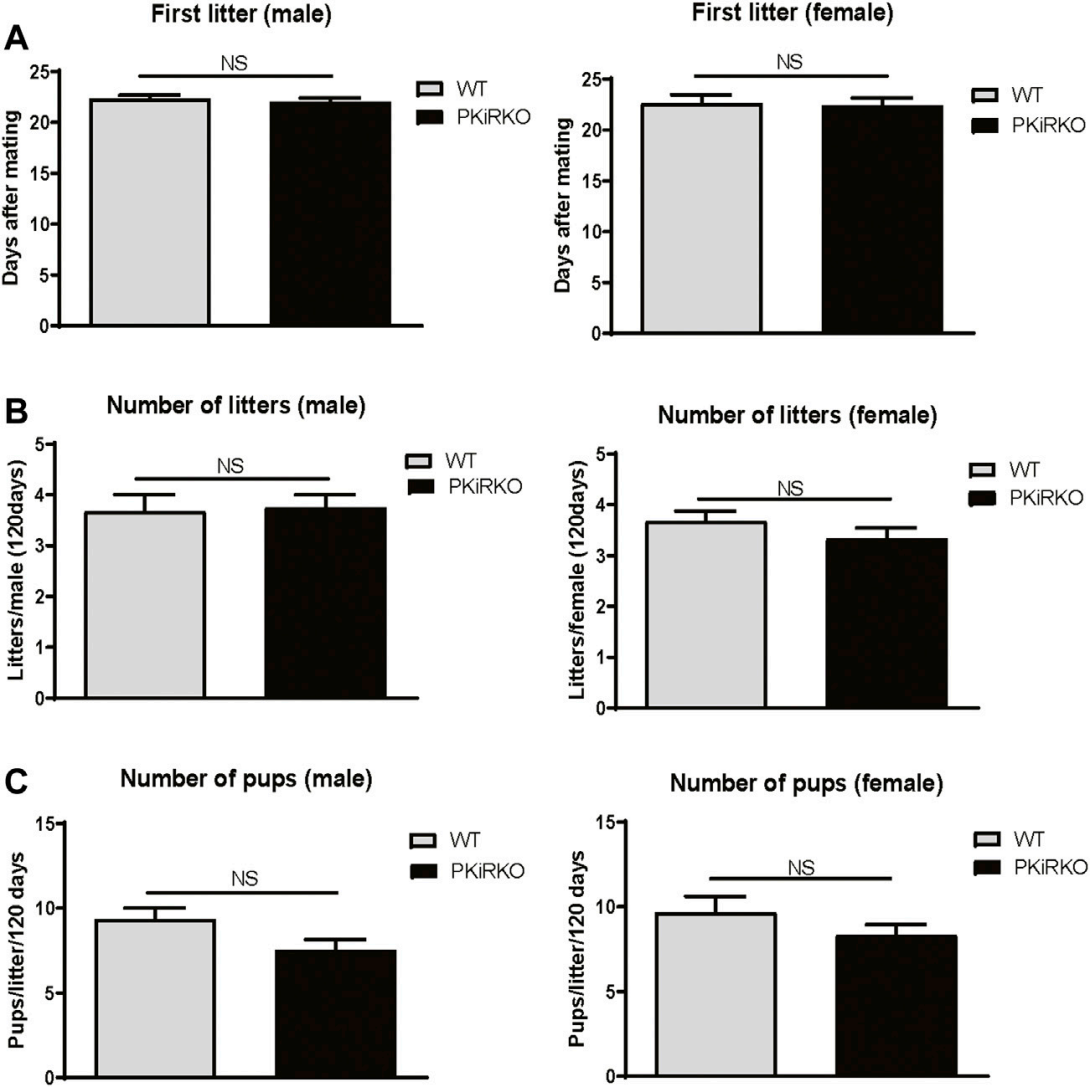
PKiRKO Mice Have Normal Fertility. **(A)** After introduction with WT female (left panel) or male (right panel) respectively, the day of first litter was recorded. Values are mean ± S.E.M. NS, no significant. **(B)** Total numbers of litters per male (left panel) or female (right panel) was not significantly different between WT and PKiRKO mice. **(C)** Number of pups per litter was also not significantly different between WT and PKiRKO mice (male, left panel; female right panel).

PKiRKO male and female mice demonstrated a normal ability to produce offspring, as shown in Figure 4 A-C. Female WT mice bore their first litter with a similar latency after introduction to PKiRKO males or WT males ((22.0 ± 0.8days, *n = 4* (PKiRKO) vs. 22.3 ± 0.6 days, *n =* 3(WT); *p >* 0.05)) (Figure 4A). Female WT mice had the same number of litters with PKiRKO and WT sires ((3.8 ± 0.5, *n = 4* (PKiRKO) vs. 3.7 ± 0.6, *n = 3* (WT); *p >* 0.05)) (Figure 4B) and a similar number of pups per litter ((7.4 ± 1.4, *n = 4* (PKiRKO) vs. 9.3 ± 1.1, *n = 3* (WT); *p >* 0.05)) (Figure 4C). Similar results were found between PKiRKO females and WT females. Female WT and PKiRKO mice bore their first litter with a similar latency after introduction to WT males ((22.4 ± 0.1 day, *n = 6* (PKiRKO) vs. 22.6 ± 0.1 day, *n =* 7(WT); *p >* 0.05)) (Figure 4A, right panel). Female WT and PKiRKO mice had the same number of litters with WT males ((3.3 ± 0.2, *n = 6* (PKiRKO) vs. 3.7 ± 0.2, *n = 6* (WT); *p >* 0.05)) (Figure 4B, right panel) and a similar number of pups per litter ((8.3 ± 0.6, *n = 6* (PKiRKO) vs. 9.7 ± 1.0, *n = 6* (WT); *p >* 0.05)) (Figure 4C, right panel).

## Discussion

Expression of KISS1R in the pituitary gonadotroph (Gutiérrez-Pascual et al., 2007) implicates the pituitary gonadotroph as a possible target for kisspeptin action, in addition to its well described role in GnRH neuron function. The functional significance of pituitary and gonadotroph KISS1R is not well defined and may be the target of either centrally or peripherally derived KISS1. We sought to assess the role of KISS1 signaling at the level of the pituitary gonadotroph in mice by the development of a gonadotroph *Kiss1* knockout model (PKiRKO). We demonstrate that near complete reduction of pituitary *Kiss1r* expression in males is associated with lower FSH levels in males with normal gonad function and reproductive function. Similar fertility results were also found between PKiRKO females and WT females. The pituitary Kiss1r signaling is not essential for reproduction. This is indirectly supported by recently published paper which showed Kiss1 rescue in the KNDy neurons restored LH pulses and follicular development in female global Kiss1 knockout rats. (Nagae et al., 2021).

Qualitative PCR analysis indicated that the pituitary gland had the most amount of *Kiss1r* expression outside of the hypothalamus (Figure 1D). *Kiss1r* mRNA expression is detectable at low levels in tissues such as ovary, liver and brown adipose tissues with defined physiological roles for Kiss1r (Song et al., 2014; Tolson et al., 2020; Ruohonen et al., 2022). Kiss1r is expressed on mouse pituitary gonadotrophs in rat (Richard et al., 2008) and sheep (Backholer et al., 2009). The PKiRKO mice had an 88% reduction in *Kiss1r* gene expression in the pituitaries of male mice and 64% in female mice. The gonadotroph comprises a minority of the pituitary cell population so kisspeptin receptor expression in other pituitary cells may account for the observed residual expression in the gonadotrope specific KO mice. Indeed, the kisspeptin receptor has been colocalized to ovine and goldfish lactotrophs and somatotrophs (Smith et al., 2008; Yang et al., 2010). The αGSU transgenic Cre mouse effectively deletes floxed genes in pituitary gonadotrophs (Brothers et al., 2010) indicating that the residual expression of *Kiss1r* in the pituitary of PKirKO may be not be in the gonadotroph.

The PKiRKO male mice had a significantly lower morning FSH level (60% of WT, Figure 3F). This lower FSH did not seem to affect reproductive function, as the PKiRKO mice also had normal testosterone, testes cell structure on gross histology, and normal testes size (Figure 3). Global null Kiss1r male mice exhibit infertility, undetectable LH and testosterone with rescue of, spermatogenesis upon testosterone supplementation. FSH levels were 10% of WT males (Goto et al., 2020). The lack of LH and testosterone together with extremely low FSH levels accounted for the differences in spermatogenesis and testicular development in these males. Mice with complete absence of FSH exhibit decreased testes size and sperm numbers and motility (Kumar et al., 1997); Therefore, there may be a threshold FSH needed for normal spermatogenesis and fertility. FSH levels in PKiRKO male mice were 60% of WT so possibly above a threshold and thus accounted for the lack of dramatic phenotype in the PKiRKO mice. However, long term effects of lower FSH on testis maybe very subtle and need to be carefully examined in the future.

Investigators have observed KISS1 induction of LH secretion in cultured primary rat and primate pituitary cells (Navarro et al., 2005; Luque et al., 2011) derived from females and up-regulation of gonadotropin gene β-subunits, LHβ and FSHβ gene expression in LβT2 cells (Witham et al., 2013; Goto et al., 2020), although others have seen no direct effect of KISS1 on LH or FSH secretion (Matsui et al., 2004; Thompson et al., 2004). Studies suggest that KISS1 may act directly on pituitary gonadotropes to stimulate LH release (Castellano et al., 2006; Gutiérrez-Pascual et al., 2007; Luque et al., 2011) or to increase gonadotropin or *Gnrhr* gene expression (Naor et al., 1980) were observed *in vitro* using cell lines or primary culture. The complex hormonal milieu of the *in vivo* pituitary may account for the lack of difference in LH and fertility between the control and PKiRKO animals in this study.

The LH and FSH response to peripheral (Matsui et al., 2004) and central (Messager et al., 2005) administration of kisspeptin is used to assess GnRH neuron activity. The LH and FSH of humans and mice with disorders of GnRH neuron causing hypogonadism do not rise after peripheral kisspeptin administration (Chan et al., 2014). These data agree with our results that there does not appear to be a critical role for kisspeptin receptor on gonadotrope regulation of LH secretion, and gonadal function.

Overall this study suggests that absence of kisspeptin signaling in pituitary gonadotrophs affects FSH levels in males without affecting testes function or fertility in both male and female mice.

## Data Availability

The original contributions presented in the study are included in the article/Supplementary Materials, further inquiries can be directed to the corresponding author.
